# Safety and efficacy of PG102P for the control of pruritus in patients undergoing hemodialysis (SNUG trial): study protocol for a randomized controlled trial

**DOI:** 10.1186/s13063-019-3753-1

**Published:** 2019-11-28

**Authors:** Yong Chul Kim, Jae Yoon Park, Sohee Oh, Jang-Hee Cho, Jae Hyun Chang, Dae Eun Choi, Jung Tak Park, Jung Pyo Lee, Sejoong Kim, Dong Ki Kim, Dong-Ryeol Ryu, Chun Soo Lim

**Affiliations:** 1 Department of Internal Medicine, Seoul National University Hospital, Seoul, Republic of Korea; 2 Department of Internal Medicine, Dongguk University Ilsan Hospital, Gyeonggi-do, Republic of Korea; 3 Department of Biostatistics, Seoul National University Boramae Medical Center, Seoul, Republic of Korea; 4 Department of Internal Medicine, Kyungpook National University School of Medicine, Daegu, Republic of Korea; 5 Division of Nephrology, Department of Internal Medicine, Kyungpook National University Hospital, Daegu, Republic of Korea; 6 Department of Internal Medicine, Gachon University Gil Medical Center, Incheon, Republic of Korea; 7 Department of Nephrology, School of Medicine, Chungnam National University, Daejeon, Republic of Korea; 8 Department of Internal Medicine, College of Medicine, Institute of Kidney Disease Research, Yonsei University, Seoul, Republic of Korea; 9 Department of Internal Medicine, Seoul National University Boramae Medical Center, 20 Boramae-ro 5-gil, Dongjak-gu, Seoul, 07061 Republic of Korea; 10 Department of Internal Medicine, Seoul National University College of Medicine, Seoul, Republic of Korea; 11 Department of Internal Medicine, Seoul National University Bundang Hospital, Seongnam, Republic of Korea; 12 Department of Internal Medicine, School of Medicine, Ewha Womans University, Seoul, Republic of Korea; 13 Tissue Injury Defense Research Center, Ewha Womans University, Seoul, Republic of Korea

**Keywords:** Uremic pruritus, Hemodialysis, PG201P

## Abstract

**Background:**

Pruritus in patients undergoing hemodialysis is a highly prevalent complication that affects quality of life. Several medications are currently used for the treatment of uremic pruritus, but these are not satisfactory. PG102P, which is prepared from *Actinidia arguta*, has an immune-modulating effect on pruritus. This trial is designed to assess the antipruritic effect of PG102P compared with placebo.

**Methods:**

This multicenter, randomized, double-blind, placebo-controlled clinical trial will include 80 patients undergoing hemodialysis. The patients will be randomized in a 1:1 ratio to a treatment group (PG102P 1.5 g/day) or a control group (placebo). The treatment will last for 8 weeks, followed by a 2-week observational period. During the observational period, all of the patients will maintain the antipruritic treatment previously used. The primary endpoint will be measured as the difference in visual analog scale between the groups before and after treatment. Secondary outcomes include serum levels of total immunoglobulin E, eosinophil cationic protein, potassium, calcium, phosphorus, intact parathyroid hormone, and blood eosinophil count between weeks 0 and 8. Kidney Disease and Quality of Life and Beck’s Depression Inventory questionnaires will be conducted. Safety assessments and any adverse events that occur will also be evaluated.

**Discussion:**

The SNUG is a clinical study that aims to investigate the antipruritic effect of PG102P to ameliorate itching in patients undergoing hemodialysis.

**Trial registration:**

Clinical Trials.gov, NCT03576235. Registered on 4 July 2018.

## Background

Pruritus is a common and distressing complication in patients with end-stage renal disease [1, 2]. A global cross-sectional study in 12 countries with more than 18,000 patients undergoing hemodialysis (HD) reported that 42% of patients experienced moderate-to-severe pruritus [3]. Pruritus is associated with impaired quality of life, sleep disturbance, depression, and increased risk of mortality [4].

The pathophysiologic mechanism of pruritus is largely unclear [5]. However, anemia, hypercalcemia, hyperphosphatemia, secondary hyperparathyroidism, and hypermagnesemia are known to be associated with uremic pruritus [6, 7]. Moreover, more than 60% of patients undergoing HD who do not have significant metabolic disorders also suffer from chronic pruritus [8].

A longitudinal study of uremic pruritus reported that changes in itching intensity more than 20% significantly improved quality of life in patients with moderate-to-severe itching symptoms [9]. However, treating patients with uremic pruritus is a challenge. Antihistamines, steroids, emollients, and phototherapy are currently used, but have not been thoroughly investigated. Adequate skin hydration is the cornerstone of antipruritic treatment because xerosis is common in patients undergoing HD. In a recent review article, gabapentin is recommended due to consistent successful results in several clinical trials [10–12], which included only 25 to 34 patients. In a recent study, oral antihistamines were used by 57% of clinicians as first-line treatment for uremic pruritus [13], but were no longer recommended in the latest reviews [14, 15]. Therefore, a novel therapeutic option is necessary to ameliorate uremic pruritus.

PG102P, which is prepared from *Actinidia arguta*, generally called hardy kiwifruit, contains an orally active immune-modulating activity through T helper 1/2 pathways [16] and by inducing regulatory T cells [17]. It shows an anti-inflammatory effect in various murine models, such as atopic dermatitis [18], allergic diarrhea [19], and asthma [20]. Therefore, we hypothesized that the immune-modulating property of PG102P would play a role in controlling itching. In this study (the SNUG trial) we aim to investigate the antipruritic effect of PG102P in comparison with placebo in 80 patients undergoing HD.

## Methods/design

### Study design

**Fig. 1:**
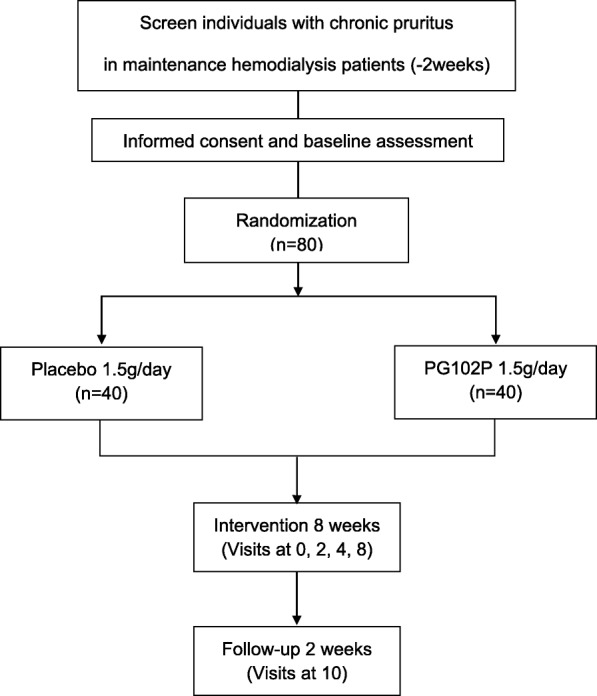
Flow chart of the SNUG trial

This is a multicenter, randomized, double-blind, placebo-controlled trial in which one group will be treated with PG102P (1.5 g/day) and the other with a placebo. It is an investigator-initiated clinical trial. The overall study algorithm is illustrated in Fig. 1. A superiority trial is planned to test the hypothesis that PG102P is effective in relieving pruritus for patients with end-stage renal disease undergoing HD. Nine tertiary university hospitals (Seoul National University Boramae Medical Center, Seoul National University Hospital, Seoul National University Bundang Hospital, Ewha Womans University Mokdong Hospital, Severance Hospital, Chungnam National University Hospital, Gachon University Gil Medical Center, Kyungpook National University Hospital, and Dongguk University Ilsan Hospital) will participate in the trial. It was retrospectively registered at http://www.clinical trials.gov/ (NCT03576235). Patients aged over 19 years who have chronic pruritus despite conventional antipruritic treatment undergoing HD will be screened for the study participation. During the study period, antipruritic drugs which had been started before this study were administered in all of the study populations without changing the dosage of the drugs.

### Inclusion criteria

The inclusion criteria include the following:

1. Age over 19 years
2. Patients with adequate HD (a dialysis efficiency calculator, or Kt/V, score >1.2, 4 h of HD three times per week).
3. Maintenance patients undergoing HD (>3 months) with chronic pruritus
4. Mean visual analog scale (VAS) score >4 for the last 5 days in the 14-day preobservation period
5. Participants who were allowed to continue the antipruritic drug treatment at the same dosage and administration schedule as used at baseline throughout the study period
6. Patients who agreed to participate in this trial and provided written informed consent

### Exclusion criteria

The exclusion criteria are outlined below:

1. Intact parathyroid hormone >1000 pg/mL within 1 month
2. Serum potassium >7.0 mg/dL
3. HIV antibody-positive
4. Aspartate transaminase (glutamic oxaloacetic transaminase) or alanine transaminase (glutamic pyruvic transaminase) >3 times the upper limit of normal
5. Scheduled to have kidney transplantation within 3 months
6. Cancer history with current treatment
7. Active infection with current treatment
8. Current itching with dermatologic diseases other than uremic pruritus
9. Pregnancy, childbearing potential during the study period, or breastfeeding
10. Allergy or hypersensitivity reaction to PG102P
11. History of participating in another clinical trial within 2 months or planning to participate in another clinical trial
12. Not eligible to participate in this trial as a decision of the researchers

### Randomization

Randomization will be performed by an independent statistician using a computerized random number generator through the block randomization method of SAS 9.3 software (SAS Institute, Cary, NC, USA). Eligible participants will be randomly assigned 1:1 to either a placebo group or a treatment group. All subjects eligible for selection/exclusion criteria at visit 2 (baseline visit, week 0) will be assigned to a group according to the allocation codes of the blocked randomization method. Each patient will be given a unique study number. An independent data manager who is not involved with the clinical practice or patient recruitment will create the randomization sequence. The practitioners, participants, outcome assessors, and statisticians will be kept blinded to treatment allocation throughout the trial. The randomization lists will be computer-generated and concealed from the investigators by an independent data manager not involved in the study. This information will remain confidential and will not be available to the researchers. In this trial, emergent unblinding is not applicable.

### Follow-up

The participants will visit the hospital seven times during the study: a screening visit (visit 1), visits during active treatment (visits 2, 3, 4, and 5), and a follow-up visit (visit 6). Each interval between visits is 2 weeks, except that between visits 4 and 5 (which is 4 weeks).

### Primary endpoint and measurement of itch

The primary endpoint is the change in VAS between visit 2 (week 0) and visits 3, 4, 5, and 6 (weeks 2, 4, 8, 10)—VAS change from baseline.

Itch severity will be measured by the patients using a VAS. A VAS consisting of a 10-cm horizontal line with 1-cm scale markings will be used. The patients will be asked to mark the intensity of their itch on the scale, with the strongest possible itch marked at the right end of the line (10 cm) and no itch marked at the left end (0 cm). The patients will be asked to mark the VAS value to record the worst degree of itch experienced during the previous day. Patients with an average VAS value of 4 or more for 5 consecutive days before visit 2 (week 0) will be enrolled. The average value of VAS measured 3 consecutive days before each visit (visits 3 to 7) will be used to evaluate itching.

### Secondary and other endpoints

Total immunoglobulin E, eosinophil count, eosinophil cationic protein, phosphorus, calcium/potassium, intact parathyroid hormone, Kidney Disease Quality of Life, and Beck Depression Inventory will also be assessed between week 0 and week 8.

### Clinical and laboratory evaluations

Physical examination, comorbidity, and medication will be reviewed. Laboratory evaluations, including complete blood cell count, serum glucose, uric acid, total protein, albumin, total bilirubin, aspartate transaminase, alanine transaminase, alkaline phosphatase, blood urea, creatinine, total cholesterol, urinalysis, urine human chorionic gonadotrophin, and HIV status, will also be performed at visit 1.

### Safety assessment and adverse events

**Fig. 2:**
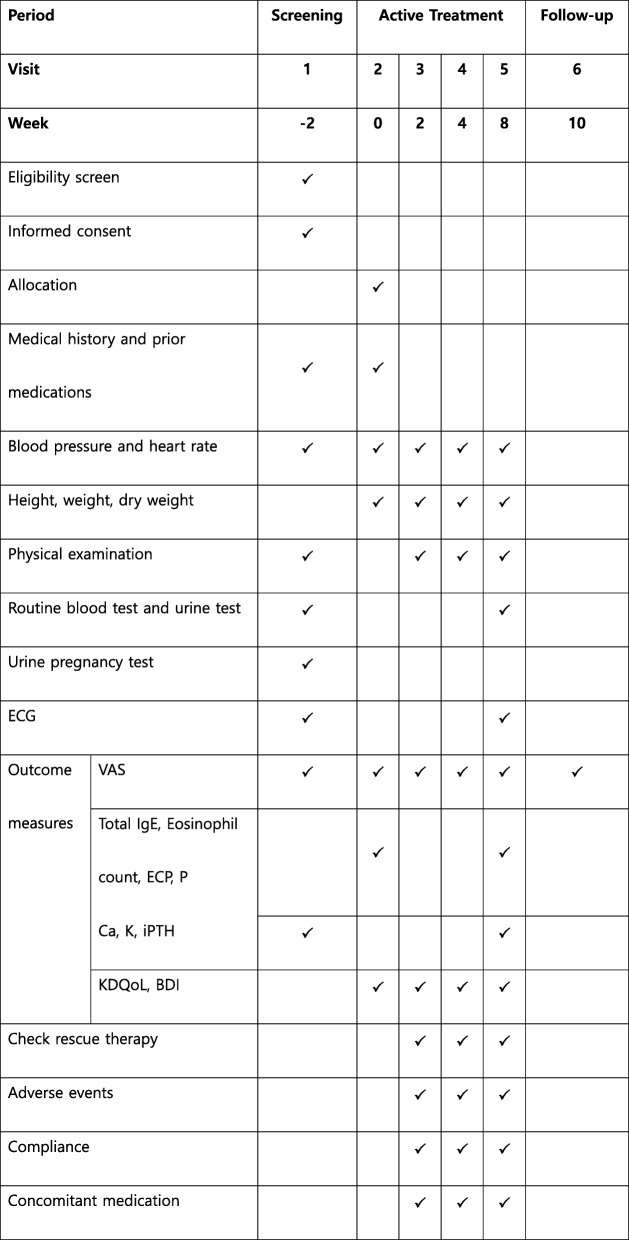
Timeline of study procedures and outcome assessments. BDI Beck Depression Inventory, Ca calcium, ECG electrocardiogram, ECP eosinophilic cationic protein, IgE immunoglobulin E, iPTH intact parathyroid hormone, K potassium, KDQoL Kidney Disease Quality of Life, P phosphorus, VAS visual analog scale

Information on adverse events (AEs) will be obtained through nondirective questioning to every participant at each visit. The safety assessments include laboratory tests (hematology/blood chemistry, urinalysis), electrocardiogram, chest x-ray, physical examination, blood pressure, pulse rate, and changes in body weight. At least once after randomization, any AEs that occur after administration of the investigational drug will be assessed. All AEs will be summarized and presented with regard to severity, relationship to the investigational drug, and outcome. The schedule of enrollment, interventions, and assessments is presented in Fig. 2.

### Sample size calculations

The enrolled participants will be randomly divided into two groups by at a 1:1 ratio. Under the assumption of 2.1 cm (with a pooled standard deviation of 2.4 cm) in the mean change difference in VAS values between the PG102P and placebo groups in a preceding pilot study with 0.9 of power, two-sided, and 0.05 of alpha, 28 participants will be allocated to each group. Considering a dropout rate of 30%, 80 participants will be needed (40 participants per group).

### Data collection and management

All data will be recorded by trained clinical investigators from each participating hospital in a standardized electronic case report form using a web-based database (Bethesdasoft Co., Ltd., http://www.mytrial.co.kr). Data will be handled confidentially and anonymously by qualified data managers. Quality control will involve collecting data on adherence to the intervention, patient inclusion and follow-up, as well as monitoring the quality of the data entry. Missing data or inconsistencies in the data will be detected by programs, and the results will be reported back to the investigator for resolution. If any given case is missing a main variable, the results of the closest observation are carried forward to the absence of the test data (last observation carried forward). Through this iterative process, we will clean the data and finally perform a database lock. All database backups of the electronic case report form will be done in real time.

### Statistical analyses

Three analysis sets will be used for analyzing data. First, a safety analysis set will be applied to analyze safety evaluation parameters. Safety analysis will be performed for a group of subjects who have ingested at least one treatment after random assignment. Second, a full analysis set will be applied, which refers to the ideal set of subjects as close as possible to the principle of intentionality analysis (including all of the subjects randomized to enroll and receive at least one treatment). Third, a per-protocol set will be applied when all of the factors are in accordance with the research plan; patients show good compliance using 80–120% of the prescribed dose. All of the baseline variables must be available, and there must be no major violation of the test plan.

For the primary endpoint, the degree of change of the VAS averaged after treatment from baseline will be analyzed using the paired *t* test or Wilcoxon signed rank test in each group. The difference between the groups will be analyzed with the two-sample *t* test or Wilcoxon rank sum test. Additionally, an analysis of covariance will be conducted with adjustments for covariates. Similar to the primary endpoint, the same analyses will be performed for the secondary endpoints. For categorical variables, the McNemar test will be carried out to evaluate the change from baseline in each group.

Demographic and clinical characteristics will be reported in terms of mean ± standard deviation for continuous variables, or as frequencies and percentages for categorical variables. Differences between groups will be analyzed using the two-sample *t* test or Wilcoxon rank sum test for continuous variables and the χ^2^ test or Fisher’s exact test for categorical variables.

AEs and adverse drug reactions will be tabulated for each treatment group in accordance with the system organ class and preferred terms of the Medical Dictionary for Regulatory Activities. The AEs and adverse drug reactions will then be classified according to severity, seriousness, and causal relationship to the study drug. Intergroup comparisons will be conducted using the χ^2^ test or Fisher’s exact test.

A value of *P* < 0.05 will be considered statistically significant. All analyses will be performed using SPSS Statistics software (v21.0; IBM Corporation, Armonk, NY, USA).

## Discussion

This study will compare the changes in VAS between the PG102P and placebo groups in patients with uremic pruritus undergoing HD. The aim of PG102P treatment is to alleviate the itching sensation to improve the quality of life, including improving depression, insomnia, and ultimately the risk of mortality of patients.

Novel antipruritic drug development and research are actively ongoing. Naltrexone, a μ-opioid receptor antagonist, was investigated in a randomized control trial (RCT), but showed no effect and was accompanied by a high incidence of gastrointestinal side effects [21].

Nalfurafine, a κ-opioid receptor agonist, has been recently proven to effectively reduce itches that were refractory to current treatments with few significant adverse reactions in an RCT with 337 patients undergoing HD [22]. Nalfurafine was approved for clinical use by the Ministry of Health, Labour and Welfare of Japan in 2009. Nalfurafine seems to be potent, but it is expensive and currently prescribed only in Japan [13].

Pregabalin was examined in an RCT with 179 patients undergoing HD and was found to reduce VAS for pruritus by 6.6 cm at 12 weeks after starting the drug [23]. This reduction is remarkably larger than that seen with nalfurafine (4.2 cm at week 24 and 4.4 cm at week 52). A large-scale RCT is warranted in the future for validation.

PG102P is a water-soluble extract prepared from the edible fruit of *A. arguta*; therefore, safety problems are not an issue. A previous toxicity study showed that PG102P is a safe agent [16]. Moreover, in an RCT with 90 asymptomatic atopic dermatitis patients in 2011, no serious AEs were observed [24]. PG102P is also inexpensive.

We introduced VAS as a monodimensional scale for assessing pruritus intensity [25]. It is a simple method and has been validated in large-scale studies consisting of chronic pruritus patients, especially in RCTs of patients with uremic pruritus [10, 21–23]. It is highly reproducible and shows great correlation between scales. However, a VAS only provides the itch intensity at a specific point in time, and is susceptible to cofounding factors such as current mood, stress and comorbidities. Multidimensional scales, such as the Itch Severity Scale [26], a self-reported seven-item scale, are an alternative method. They provide comprehensive scores of itch intensity, sleep disturbance and burden by the symptom, but are not established in clinical practice.

Although we calculated the adequate sample size, the absolute number is quite small. The sample could potentially include relatively homogeneous patients so there might be a limitation in generalizing the results of our RCT to entire HD patient populations. Compared with cross-sectional designs, repeated measures designs allow the definitive evaluation of within-person changes over time and have higher statistical power for detecting differences. Thus, fewer participants are required for conducting a study. Most of the RCTs on uremic pruritus have traditionally used repeated measures designs [27].

In summary, the SNUG study is a multicenter, randomized, double-blind, placebo-controlled clinical trial that will evaluate the efficacy and safety of PG102P versus placebo in patients with chronic pruritus undergoing HD. This study is expected to confirm whether PG102P has an antipruritic effect.

## Trial status

Patient recruitment began on 1 May 2018. At the time of manuscript submission, 45 patients had been recruited, and recruitment will be completed by 31 December 2018.

## Supplementary information

**Additional file 1.** SPIRIT 2013 checklist.

## Data Availability

Trial information can be found at ClinicalTrials.gov, NCT03576235. A completed SPIRIT checklist and figure are available in Additional file 1. The datasets generated and/or analyzed during the present study are available from the corresponding author on reasonable request.

## Abbreviations

AE: Adverse event
HD: Hemodialysis
RCT: Randomized controlled trial
VAS: Visual analog scale
